# Factors associated with exclusive breastfeeding practices among mothers in Goba district, south east Ethiopia: a cross-sectional study

**DOI:** 10.1186/1746-4358-7-17

**Published:** 2012-11-27

**Authors:** Tesfaye Setegn, Tefera Belachew, Mulusew Gerbaba, Kebede Deribe, Amare Deribew, Sibhatu Biadgilign

**Affiliations:** 1 College of Health Sciences, Department of Nursing, Madawalabu University, Bale Goba, Ethiopia; 2 College of Public Health and Medical Sciences, Department of Population and Family Health, Jimma University, Jimma, Ethiopia; 3 College of Public Health and Medical Sciences, Department of Epidemiology and Biostatistics, Jimma University, Jimma, Ethiopia

## Abstract

**Background:**

Exclusive breastfeeding is defined as feeding infants only breast milk, be it directly from breast or expressed, with no addition of any liquid or solids apart from drops or syrups consisting of vitamins, mineral supplements or medicine, and nothing else. Several studies have shown that exclusive breastfeeding for the first six months plays a great role in preventing morbidity and mortality. However, in Ethiopia a large portion of infants are not exclusively breastfed according to the infant feeding recommendations. Understanding the factors that influence exclusive breastfeeding is crucial to promoting the practice. This study was carried out to identify factors predicting exclusive breastfeeding among mothers in Bale Goba district, south east Ethiopia.

**Methods:**

A community-based cross-sectional study was conducted from March to February 2010 involving both quantitative and qualitative data. A total of 608 mothers were selected randomly. A convenience sampling technique was used to generate the qualitative data. The qualitative data were analyzed using thematic frameworks. A multivariable logistic regression analysis was used to identify independent predictors of exclusive breastfeeding after controlling for background variables.

**Results:**

The prevalence of exclusive breastfeeding in the last 24 hours preceding the survey was 71.3%. The median duration of exclusive breastfeeding was three months and mean frequency of breastfeeding was six times per day. Being unemployed [AOR: 10.4 (95% CI: 1.51, 71.50)] and age of infants of less than two months [AOR: 5.6 (95% CI: 2.28, 13.60)] were independently associated with exclusive breastfeeding.

**Conclusions:**

A large proportion of infants are not exclusively breastfed during the first 6 months, despite what is recommended in the national and global infant and young child feeding (IYCF) guidelines. Employed mothers were less likely to practice exclusive breastfeeding, implying the need for promoting workplace breastfeeding practices and creating an enabling environment for exclusive breastfeeding. Extensions of maternity leave up to the first six month of child’s age to achieve optimal level of exclusive breastfeeding practices should also be looked into as an alternative solution.

## Background

The World Health Organization (WHO) recommends exclusive breastfeeding for the first six months of life and continued breastfeeding up to two years of age or beyond. Promotion of exclusive breastfeeding is the single most cost-effective intervention to reduce infant mortality in developing countries [1-5]. It is estimated that sub-optimal breastfeeding, especially non-exclusive breastfeeding in the first six months of life, results in 1.4 million deaths and 10% of diseases in under-fives. Non–exclusive breastfeeding also has long term impact, including poor school performance, reduced productivity, and impaired intellectual and social development. It can also increase the risk of dying due to diarrhea and pneumonia among 0–5 month old infants by more than two-fold [2,3].

Evidence shows that of the sixty percent of under-five mortality caused by malnutrition (directly or indirectly), more than two-thirds of those are associated with inappropriate breastfeeding practices during infancy. Not more than 35% of infants worldwide are exclusively breastfed during their first four months of life [2,3,5]. There is a wide range of variation in the practice of exclusive breastfeeding among developing countries, with the rates documented being: Brazil (58%), Bangalore (40%), Iran (Zahedan) (69%), Iran (28%) Beruwala (Kalutara) (15.5%), Lebanon (10.1%), Nigeria (20%), Bangladesh (34.5%), Jordan (77%) [6-15]. In Ethiopia, 49% of infants were exclusively breastfed for the first six months, while 56.9% were exclusively breastfed for the first four months [11,16,17].

Cognizant of the high prevalence of inappropriate child feeding practices and the importance of exclusive breastfeeding, the Ethiopian government developed the Infant and Young Child Feeding (IYCF) guideline in 2004 [18]. Since then, varying levels of interventions, giving due emphasis to key messages of exclusive breastfeeding, were being given both at health institution and community level. Nonetheless, these efforts were not based on organized evidence on the level of existing practices, which might be due to lack of studies which explored the factors predicting the low proportion of exclusive breastfeeding. There are no studies that examined and documented the magnitude and associated factors of exclusive breastfeeding in the study area. The objective of this study is, thus, to assess factors associated with exclusive breastfeeding among mothers in Bale Goba district, Southeast Ethiopia.

## Methods

### Study setting and participants

A community-based cross-sectional study, using on both quantitative and qualitative methods of data collection, was conducted in Bale Zone, Goba district, Southeast of Ethiopia. Bale Zone is the second largest zone in Oromia regional state in Ethiopia, with an area of 67.329.6 km^2^ and is located 430 km from the capital, Addis Ababa. The temperature ranges from 3.5-32°c. Goba district is one of the 20 districts (*Woreda*) in Bale Zone, having both rural and urban populations. The district has one hospital, one health center and more than 20 health posts [19].

The sample size for this study was determined using a formula for estimation of single population proportion assuming an expected prevalence for exclusive breastfeeding of 50%, 95% confidence level, 5% margin of error, a design effect of 2 and a non-response rate of 10%. A total of 668 mother-infant pairs were identified using stratified sampling technique from the urban and rural residences. Then a census was conducted to get the sampling frame for selecting mother-infant pairs by simple random sampling technique. For the in-depth interview, 23 individuals (6 health care providers, 9 breastfeeding mothers from urban and rural areas and 8 community health extension workers) were selected using a convenience sampling technic.

### Measurements

Quantitative data were collected using a validated questionnaire adapted from the Ethiopian Health and Demographic Survey (EDHS), WHO and LINKAGE project which were designed to assess infant and young child feeding practices in developing countries including Ethiopia [2,7,11,20]. The questionnaire was translated and contextualized to the local situation. Data on breastfeeding practices, socio-demographic factors, obstetric factors such as birth intervals, parity, and antenatal care visits and health service related factors/practices including pre/postnatal counseling were collected by interviewing the mothers of index children. A semi-structured open-ended interview guide was used as a guide for the key informant interview. The data were collected by 12th grade complete students who took an intensive training for two days on the questionnaire and on general approaches to data collection. The WHO definition of exclusive breastfeeding: the other/care giver reported that nothing else but breast milk was given in the last 24 hours preceding the interview, was used [2]. Exclusive breastfeeding was measured by asking mothers with infants aged between 0 and 6 months to provide information about the history of infant feeding for the last 24 hours. The prevalence of exclusive breastfeeding was calculated as the ratio of infants below 6 months who fed only on breast milk in the 24-hours preceding the survey to the total number of children in the same age group (< 6 months of age) [20]. Respondents were also requested to answer “*For how many months did they feed their child with breast-milk only?’ This was* used to calculate the median durations of exclusive breastfeeding practice.

### Statistical analysis

Quantitative data were entered, coded, and analyzed using SPSS for windows version 16.0 (SPSS Inc. version 16.1, Chicago, Illinois). Descriptive statistics was computed to determine the prevalence of exclusive breastfeeding. Proportions were compared by exclusive breastfeeding using Pearson’s chi-square test of independence. To identify associated factors, first a bivariate logistic regression was performed for each independent variable with the outcome of interest (exclusive breastfeeding). Finally, multivariable logistic regression was done to determine independent predictors of exclusive breastfeeding. All tests were two-sided and p < 0.05 was considered statistically significant. The qualitative data were transcribed from Amharic to English language and text analysis was done manually. Data captured using the field notes were transcribed into English texts by the principal investigator. The transcribed data were read carefully, categorized and summarized by thematic areas (thematic framework analysis). The interview data were triangulated with the quantitative data.

Ethical clearance was received from the institutional review board (IRB) of Jimma University Ethical Clearance Committee (Ref. No.JURPGC/46/2010). Official letter of co-operation was also obtained from Oromia Health Bureau, Zonal Health Desk & Woreda Health Office. Informed verbal consent was secured from study participants in their own language after explaining the purpose of the study, potential risks and benefits of partaking in the study, and the right to withdraw from the study at any time. The participants were also assured about the confidentiality of the data.

## Results

From the total of 668 mother-infant pairs, 608 were included in the analysis, making the response rate 91.0%. The mean (± SD) age of mothers was 26.5 (± 5.5) years. Sixty-nine percent of respondents were Muslims by religion. The largest ethnic group was Oromo (89.1%) followed by Amhara (9.9%). Pertaining to the educational status of mothers, (61.0%) had attended formal school of which (45.2%) completed primary school (grade 1 to 8). The majorities (95.7%) of the mothers were married and (82.3%) were housewives by occupation (Table 1)*.*

**Table 1:** Socio-demographic characteristics of breastfeeding mothers in Goba District, Bale Zone, March- February 2010

**Socio-demographic variables**		**Number**	**Percent**
Age of mothers (years)	15-19	43	7.1
	20-24	173	28.5
	25-29	219	36.0
	30+	173	28.5
	Mean(SD)	26.5(±5.5)	
Religion	Muslim	422	69.4
	Orthodox	173	28.5
	Protestant	13	2.1
Ethnicity	Oromo	542	89.1
	Amhara	60	9.9
	Other*	6	1.0
Educational status of mothers	Illiterate	139	22.9
	Able to read/Write^¥^	98	16.1
	Primary(1–8)	275	45.2
	Secondary and above(9+)	96	15.8
Marital status	Married	579	95.7
	Never married	10	1.7
	Other^†^	11	1.8
Occupation of mothers	House wife	498	81.9
	Farmer	42	6.9
	Business Woman**	25	4.1
	Student	15	2.5
	Employed	13	2.1
	Other^♠^	15	2.5
Place of residence	Urban	120	19.7
	Rural	488	80.3
Sex of infant	Male	318	52.3
	Female	290	47.7
Age of infants	< 6 months	283	46.9
	> 6 months	321	53.1

*Tigre, Gurage ^†^Widowed, divorced, separated ^♠^ Daily laborer, house servants.

**A woman who is involved in some form of trading activities.

^¥^ Attended informal education and able to read and write.

**Figure 1:**
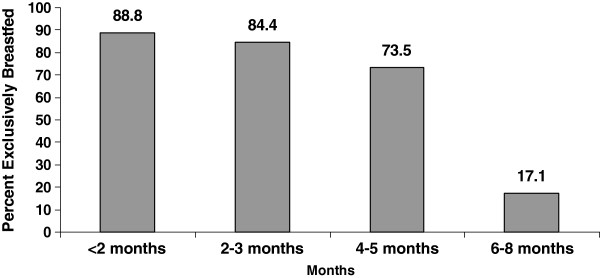
Month-specific lifetime exclusive breastfeeding among mothers in Goba District, March–February 2010.

From the total mothers who had ever breastfed their infant (98.7%), about 96.3% of them were breastfeeding at the time of the survey. The prevalence of exclusive breastfeeding for infants’ aged less than six months in the study area was 71.3% as measured by last 24 hours recall period preceding the survey date. The median duration of exclusive breastfeeding for infants less than six months was 3 months. The median frequency of exclusive breastfeeding for infants less than six months per day was 6. The results of month-specific lifetime exclusive breastfeeding analysis showed that the majority 88.8% of infants were breastfed exclusively for 2 months, while 84.4% of infants were breastfed exclusively to 2 to 3 months of age (Figure 1). For mothers with infants older than six months, 68.2% reported giving breast milk with additional food, such as cow’s milk (57.0%), cereal-based fluids (45.2%), and tea (23.9%) before their infant reached six months.

The in-depth interviews identified that lack of knowledge was the main barrier of exclusive breastfeeding. For instance, a health extension worker expressed that mothers do not consider breast milk as adequate and important:

*“… despite our effort, most mothers do not practice exclusive breastfeeding. They provide infants with some food other than breast milk. They sometimes consider breast milk as if it is not food and sufficient…they use it for appeasement when the child cries”* [Health extension worker, Lashkona kebele.] 

Lack of knowledge on exclusive breastfeeding was also substantiated by one mother with a one month old male infant, Goba town who stated:

“ . . . .I planned to feed my baby only breast milk till his six months. But I do not know how many times to feed him within a day.”

The chi-square test showed that employment status of mothers, age of infant, prelacteal feeding, parity of mothers and timely initiation of breastfeeding were significantly associated with exclusive breastfeeding (Tables 2 and 3). The binary logistic regression analysis showed that unemployed mothers were about 5 times more likely to breastfed exclusively as compared to employed mothers (OR: 5.3; 95% CI: 1.3, 21.8). Infants in the age group less than two months were 2.7 times more likely to breastfed exclusively as compared to those infants in the age group 4 to 5 months (OR: 2.7; 95%CI: 1.40, 5.13). Mothers who initiated breastfeeding within one hour of birth were 2 times more likely to practice exclusive breastfeeding than mothers who initiated after one hour (OR: 1.8; 95%CI: 1.06, 3.04) (Table 4).

**Table 2:** Chi-square tests of exclusive breastfeeding by their socio-demographic characteristics among mothers in Goba District using 24 hour recall, March–February 2010

**Variables**	**EBF**	**Non-EBF**	**P-value**
	**N (%)**	**N (%)**	
Age of mothers			
15–19	17 (73.9)	6 (26.1)	0.093
20–24	62 (74.7)	21 (25.3)	
25–29	70 (70.0)	30 (30.0)	
30–35	35 (81.4)	8 (18.6)	
35+	15 (50.0)	15 (50.0)	
Educational Status			
Illiterate	46 (76.7)	14 (23.3)	0.580
Read/Write only^¥^	122 (68.9)	55(31.1)	
Primary(1–8)	97 (71.3)	39(28.7)	
Grade 9 and above	30 (65.2)	16(34.8)	
Employment			
Employed	3 (33.3)	6 (66.7)	0.018^*^
Unemployed	196 (72.6)	74 (27.4)	
Residence			
Urban	37 (63.8)	21 (36.2)	0.154
Rural	162 (73.3)	59 (26.7)	
Sex of infant			
Male	107 (71.3)	43 (28.7)	0.998
Female	92 (71.3)	37 (28.7)	

*Fisher’s exact test and statistically significant at P < 0.05.

^¥^ Attended informal education and able to read and write.

^**§**^*EBF-* Exclusive breastfeeding.

**Table 3:** Chi- square tests of exclusive breastfeeding among mothers by their obstetric and health service-related factors among mothers in Goba District using 24 hour recall

**Variable**	**EBF N (%)**	**Non-EBF N (%)**	**P-value**
Parity of mothers			
1	49 (64.5)	27 (35.5)	
2-4	97 (77.6)	28 (22.4)	0.124
5 and more	50 (70.4)	21 (29.6)	
Birth interval(year)			
1	8 (61.5)	5 (38.5)	0.534
2-3	84 (74.3)	29 (25.7)	
4 and above	107 (69.9)	46 (30.1)	
ANC visit			
Yes	166 (71.9)	65 (28.1)	0.889
No	27 (73.0)	10 (27.0)	
No. ANC visit			
1	10 (58.8)	7 (41.2)	0.349
2-3	96 (75.0)	32 (25.0)	
= 4	88 (71.0)	36 (29.0)	
Mother given information/advice on BF at ANC visit			
Yes	76 (73.8)	27 (26.2)	0.487
No	123 (69.9)	53 (30.1)	
Place of delivery			
Home	139 (74.7)	47 (25.3)	0.305
Health institution	57 (67.9)	27 (32.1)	
Type of delivery			
Normal/Vaginal	186 (72.7)	70 (27.3)	0.605
Caesarean section	6 (60.0)	4 (40.0)	
Mother received information/advice on BF at PNC			
Yes	91 (70.5)	38 (29.5)	0.892
No	108 (72.0)	42 (28.0)	
Timely initiation of BF			
Yes	111 (77.1)	33 (22.9)	0.039*
No	88(65.2)	47(34.8)	

*significant at p-value <0.05.

**Table 4:** Multivariable logistic regression analysis showing factors associated with exclusive breastfeeding among mothers in Goba District, 2010

**Variable**	**Exclusively breastfed for the first 6 months**		**Crude odds ratio(95%C.I)**	**Adjusted odds ratio(95%C.I)**
	**Yes**	**No**		
Age of mother (years)				
15-19	17 (73.9)	6 (26.1)	1.0	
20-24	62 (74.7)	21 (25.3)	1.042(0.40,3.00)	
25-29	70 (70.0)	30 (30.0)	0.824(0.30,2.30)	
30-35	35 (81.4)	8 (18.6)	1.544(0.50,5.20)	
35+	15 (50.0)	15 (50.0)	0.353(0.11,1.14)	
Employment status				
Employed	3(33.3)	6(66.7)	1.0	1.0
Unemployed	196(72.6)	74(27.4)	5.3(1.3,21.8)*	10.4(1.51,71.50)*
Residence				
Urban	37(63.8)	21(36.2)	1.0	
Rural	162(73.3)	59(26.7)	1.6(0.8,2.88)	
Age of infant (months)				
<2	94(83.2)	19(16.8)	2.7 (1.40,5.13)*	5.6(2.28,13.60)*
2-3	28(77.8)	8(22.2)	1.9(0.78,4.62)	2.3(0.77,7.10)
4-5	61(64.9)	33(35.1)	1.0	1.0
Pre-lacteal feeding				
Yes	25(58.1)	18(41.9)	1.0	
No	159(76.4)	49(23.6)	2.391.18,4.64)*	
Mode of delivery				
Vaginal/Normal	186(72.7)	70(27.3)	1.7(0.5,6.5)	
C/S	6(60.0)	4(40.0)	1.0	
Parity of respondent				
1	49(64.5)	27(35.5)	1.0	
2-4	97(77.6)	28(22.4)	1.9(1.02,3.58)*	
5 and above	50(70.4)	21(29.6)	1.3(0.65,2.62)	
Birth interval (years)				
1	8(61.5)	5(38.5)	1.0	
2-3	84(74.3)	29(25.7)	1.8(0.55,5.97)	
4 and above	107(69.9)	46(30.1)	1.4(0.45,4.7)	
No. ANC visit				
1	10(58.8)	7(41.2)	1.0	
2-3	96(75.0)	32(25.0)	2.1(0.74,5.97)	
> = 4	88(71.0)	36(29.0)	1.7(0.60,4.85)	
Advice/information at ANC				
Yes	76(73.8)	27(26.2)	1.21(0.70,2.10)	
No	123(69.9)	53(30.1)	1.0	
Advice/information at PNC				
Yes	91(70.5)	38(29.5)	1.1(0.64,1.81)	
No	108(72.0)	42(28.0)	1.0	
Timely initiation of BF				
Yes	111(77.1)	33(22.9)	1.8(1.06,3.04)*	
No	88(65.2)	47(34.8)	1.0	

* Statistically significant variables at P <0.05, C.I- Confidence Interval.

On multivariable logistic regression analyses, maternal employment status and age of infants were significant predictors of exclusive breastfeeding. The adjusted odds of unemployed mothers practicing exclusive breastfeeding was 10.4 times the odds of employed mothers (AOR: 10.4; 95%CI: 1.51, 71.50) and those infants whose age was < 2 months were 5.6 times more likely to be breastfed exclusively compared with infants in the age range of 4 to 5 months (AOR: 5.6; 95% CI: 2.28, 13.60) (Table 4).

## Discussion

This study aimed to determine the prevalence of exclusive breastfeeding, including associated factors. Ninety-eight percent of mothers had ever practiced breastfeeding which is almost similar to the national and Oromia regional ever breastfeeding rate (96%) (94%) respectively [11,17]. This study revealed that the prevalence of exclusive breastfeeding practice for infants less than six months old was 71.3%. This finding is similar to other countries such as Jordan (77%), Madagascar, (70%), Zambia, (74%), Ghana, (79%) and Bolivia (65%). This is also similar with findings in Amhara Region, (81%), Oromia Region, (62%) and South Nations and Nationalities Peoples Region (64%). But this finding is higher than the findings in Lebanon (10%), Bangladesh (36%) and the national exclusive breastfeeding prevalence in Ethiopia (49%) [7,11,17,21,22]. The median duration of exclusive breastfeeding for infants less than six months was three months. The median duration of exclusive breastfeeding in Ethiopia was documented with a wide range of variety from lowest (0.4 month for Afar Region) through the highest (4.3 months for Amhara region). Month-specific lifetime exclusive breastfeeding was assessed for those mothers with infants above six months who are currently breastfeeding and fed their infant nothing other than breast milk til six months of age. The majority (89%) of infants less than 2 months were breastfed exclusively, dropping to 17% when infants were 4–5 months of age. This finding is higher than the Ethiopian national month-specific exclusive breastfeeding rate of 67% for infants < 2 months and 32% for infants aged 4–5 months [11]. Maternal educational status and exclusive breastfeeding did not show any significant association. This is contrary to the result obtained from the Ethiopian demographic health survey, which indicated a declining trend of exclusive breastfeeding practice with the higher maternal education status [6,17].

The multivariable logistic regression analysis showed that age of infant was a predictor of exclusive breastfeeding practice. Infants in the age group < 2 months were about 6 times more likely to be exclusively breastfed when compared to infants in the age group 4–5 months. Infants in the age group 2–3 months were 2 times more likely to breastfeed exclusively when compared to those infants in the age group 4–5 months. As the age of the children approached 6 months, the rate of exclusive breastfeeding decreased significantly, which is similar to studies conducted in Iran, Uganda, Sudan, and Ethiopia [17,23-25]. This might be due to the fact that post-partum care is traditionally given in the first few months after birth where mothers remain at home, creating a chance to exclusively breastfeed their infant. The other possible reason might be that mothers might have introduced complementary feeding for their infants due to the assumption that breast milk alone would not satisfy their needs as the infant gets older. As the age of the child increased, the rate of EBF decreased significantly, which is again in conformity with reports of studies done in Uganda, Pakistan and India [26-28]. This could probably be explained by the short birth interval/spacing and other economic factors. It can also be attributed to the fact that post partum care traditionally is given in the first few months when mothers are confined at home, creating an opportunity to exclusively breastfeed their child.

This study has indicated a significant difference among employed and unemployed mothers with regard to exclusive breastfeeding (33% vs 73%) and also revealed that unemployment of the mothers is a predictor of exclusive breastfeeding, which is consistent with the findings of several studies [29-32]. This might be explained by the fact of less maternity leave (two months after delivery in our context), which makes employed mothers have less opportunity to stay at home, compromising exclusive breastfeeding. Mothers also may have to leave their babies to search for a job. These findings call for policy arguments to initiate breastfeeding-friendly work environments, as well as the extension of maternity leave to encourage mothers to exclusively breastfeed their babies to improve child health outcomes [33].

This study can be interpreted in light of its strengths and limitations. The use of validated questionnaires, both quantitative and qualitative methods of data collection and data triangulation were the strengths of this study. However, the 24-hour recall to determine exclusive breastfeeding practice means some infants who were given other liquids regularly may not have received them in the last 24 hours before the survey, which may cause overestimation of the proportion exclusively breastfed. Similar findings were also observed in several studies, showing that the 24-hour recall method can overestimate the actual EBF rate in a population study and the one-day assessment overestimated exclusive breastfeeding rates among infants younger than 4 months. Similar findings were obtained in an analysis of the Ethiopia Demographic and Health Surveys 2000, where even larger discrepancies were found among children 4–6 months old between the 24-hour recall and the 7-day recall method [34]. Several authors have questioned the validity of the 24-hour recall method [35,36]. The major criticism of the 24-hour recall method is that it misclassifies too many mothers as exclusively breastfeeding [37,38]; a proportion of mothers may be providing substances other than breast milk on an irregular, not daily, basis. Many studies have shown that a large proportion of infants who were exclusively breastfed in the previous 24 hours were either not exclusively breastfed during the previous seven days, and/or, not exclusively breastfed since birth [26,38,39]. Median duration can also be affected by maternal recall, which might be prone to recall and social desirability bias. Therefore, readers are recommended to take this into account during interpretation of these findings. There are also unusually large odds ratios and a wide confidence interval observed in this study. In addition, there are also some variables that were not significantly associated (however known in several studies) with the outcome of interest which might affect the precision. This might be due to the sample size, which might not be adequate to justify the relationships between the explanatory variables and outcome of interest, and the observed counts are also so small in some of the cells making the odds ratios so large and so wide. Therefore, any interpretation of this finding should take into account the degree of precision. In addition, this study used a cross-sectional study design, making it is difficult to establish causal associations. The fact that this study did not assess individual factors, including knowledge and attitude of mothers, as well as variables related to family and peers, are the limitations of our study.

## Conclusions

The study revealed that the prevalence of exclusive breastfeeding using 24-hour recall method was suboptimal. In this study, the duration and frequency of exclusive breastfeeding were below the World Health Organization and national infant and young child feeding recommendations. Working mothers were more likely not to exclusively breastfeed their babies. Promotion of exclusive breastfeeding through creating an enabling, breastfeeding-friendly working environment for working mothers is recommended. In addition, advocacy efforts targeting the extension of maternity leave up to the first six months after delivery should be exerted to prevent sub-optimal exclusive breastfeeding and associated health problems among children.

## Competing interests

The authors declare that they have no competing interests.

## Authors’ contributions

TS conceived and designed the study, performed analysis and interpretation of data and drafted the manuscript. MG assisted with the design conception, analysis and interpretation of data, and the critical review of the manuscript. TB assisted the study design, data interpretation, and critically reviewed the manuscript. SB, KD and AD assisted in interpretation of data, manuscript preparation and critically reviewed the manuscript. All authors read and approved the final manuscript.
